# Coats plus syndrome: a rare cause of severe gastrointestinal tract bleeding in children – a case report

**DOI:** 10.1186/s12887-022-03140-5

**Published:** 2022-03-08

**Authors:** Selcen Bozkurt, Ayse Merve Usta, Nafiye Urganci, Nida Gulderen Kalay, Gulsen Kose, Evrim Ozmen

**Affiliations:** 1 Department of Pediatrics, Karadeniz Eregli State Hospital, Zonguldak, Turkey; 2 Department of Pediatric Gastroenterology, Sisli Hamidiye Etfal Training and Research Hospital, University of Health Sciences, Istanbul, Turkey; 3 Department of Pediatrics, Sisli Hamidiye Etfal Training and Research Hospital, University of Health Sciences, Istanbul, Turkey; 4 Department of Pediatric Neurology, Sisli Hamidiye Etfal Training and Research Hospital, University of Health Sciences, Istanbul, Turkey; 5 Department of Pediatric Radiology, Sisli Hamidiye Etfal Training and Research Hospital, University of Health Sciences, Istanbul, Turkey

**Keywords:** Coats plus syndrome, Gastrointestinal bleeding, Portal hypertension, Octreotide, Leukocoria

## Abstract

**Background:**

Coats plus syndrome, cerebroretinal microangiopathy with calcifications and cysts, is a rare disease with autosomal recessive pattern occurring due to a mutation in CTC1, encoding conserved telomere maintenance component 1, gene. Besides retinal involvement, abnormalities in brain and osteopenia, serious life-threatening bleeding in gastrointestinal tract and portal hypertension can be observed.

**Case presentation:**

A 6-year-old girl with Coats plus syndrome presented to the pediatric emergency department with vomiting blood and blood in stool. An upper and lower gastrointestinal endoscopy revealed esophageal varices and vascular telangiectasia in the pyloric antrum, duodenum, and colon. She received palliative care and the bleeding was stopped after receiving intravenous octreotide. She then was followed in the pediatric gastroenterology, neurology, and ophthalmology clinics. She was later hospitalized and admitted to the intensive care unit as she continued to have intermittent gastrointestinal system bleeding. She eventually died due to severe gastrointestinal system bleeding.

**Conclusions:**

Coats plus syndrome can lead to life-threatening gastrointestinal bleeding and portal hypertension. As Coats plus syndrome is quite rare, there is little published data on this syndrome. This report presents a case of Coats plus syndrome as a rare cause of gastrointestinal bleeding and portal hypertension.

## Background

Coats plus syndrome, cerebroretinal microangiopathy with calcifications and cysts (CRMCC), is a rare disease with an autosomal recessive pattern caused by a mutation in the encoding conserved telomere maintenance component 1 (CTC1) gene. The CTC1 gene is located on chromosome 17p13.1 and plays an important role in telomere replication [1–3]. It is a multisystem disorder characterized by retinal telangiectasia and subretinal exudate, intracranial calcification, leukodystrophy, prenatal and postnatal growth retardation. In addition, parenchymal brain cysts, osteopenia with predisposition to fractures, serious life-threatening bleeding in gastrointestinal tract and portal hypertension can be observed in the patients with CRMCC [1–3]. In this case report, Coats plus syndrome, a rare cause of severe gastrointestinal bleeding and portal hypertension is presented.

## Case presentation

A 6-year-old girl presented to the pediatric emergency department with the chief complaints of vomiting blood and blood in stool. Her mother reported that she noticed the blood in her daughter’s stool three days ago and her daughter then started vomiting blood and the symptoms became worse dramatically.

**Fig. 1:**
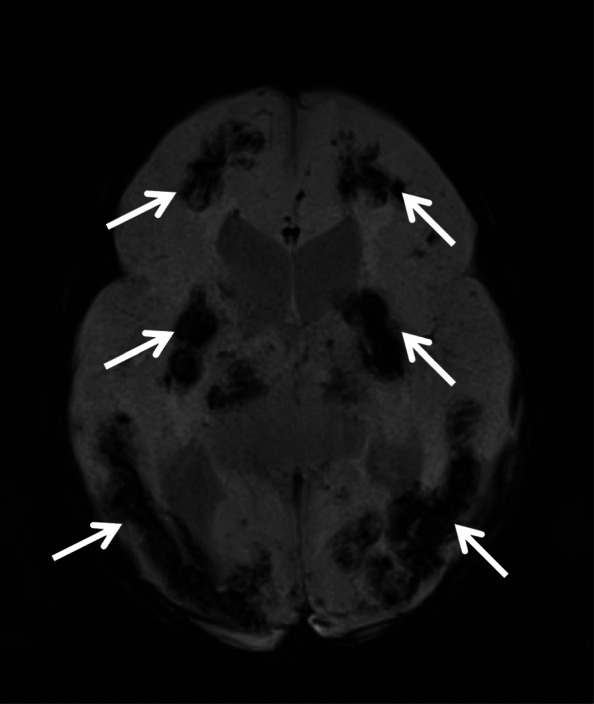
Diffuse symmetric calcifications in MRI

**Fig. 2:**
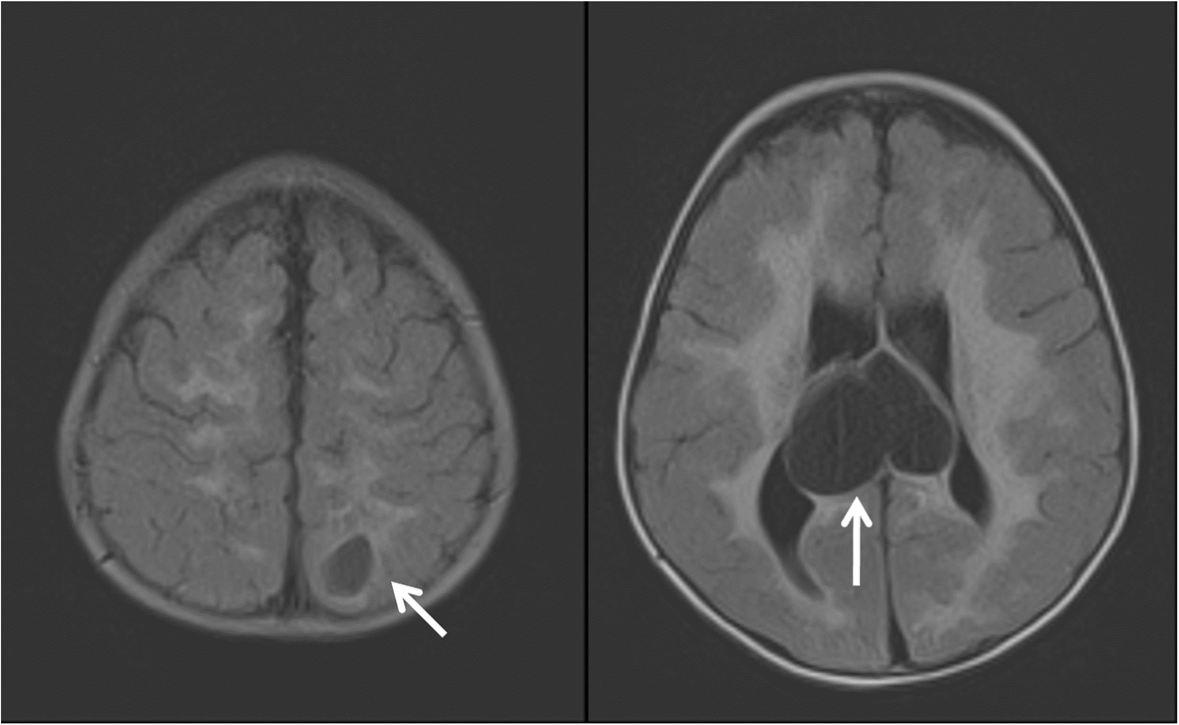
Intracranial cysts in cranial MRI

**Fig. 3:**
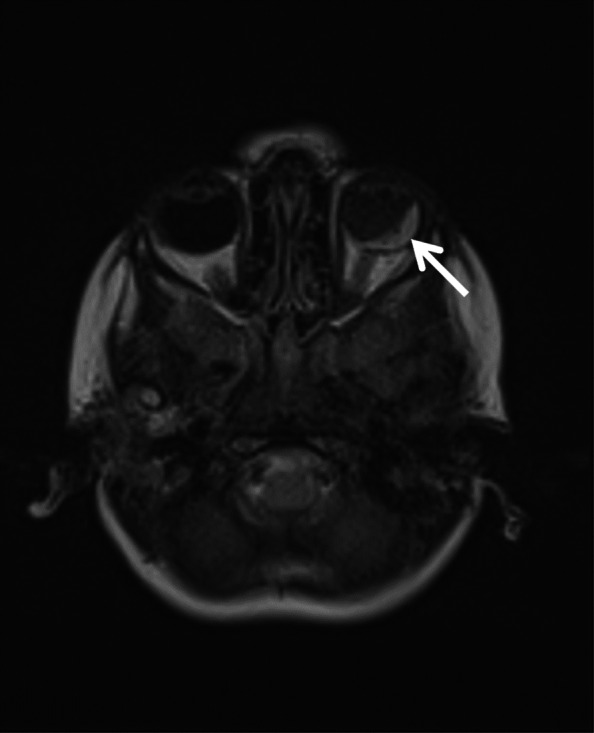
Hemorrhage in left eye in cranial MRI

The baby was delivered at 34 weeks gestation with cesarean section with intrauterine growth restriction (IUGR), hospitalized in a neonatal intensive care unit for 1 month and treated with laser photocoagulation due to the vision loss in the left eye. The cranial magnetic resonance imaging (MRI) was performed at the age of 6 months in a pediatrics neurology clinic due to convulsions and it revealed diffuse symmetric calcifications, changes suggesting hemorrhage, dilated lateral ventricles, septated cystic lesions in the area extending from the roof of the third ventricle to the lateral ventricle and also hemorrhage in the globe in left orbit (Figs. 1, 2 and 3). After further ophthalmoscopic tests, cranial findings and genetic tests, the patient was diagnosed with Coats plus syndrome.

**Fig. 4:**
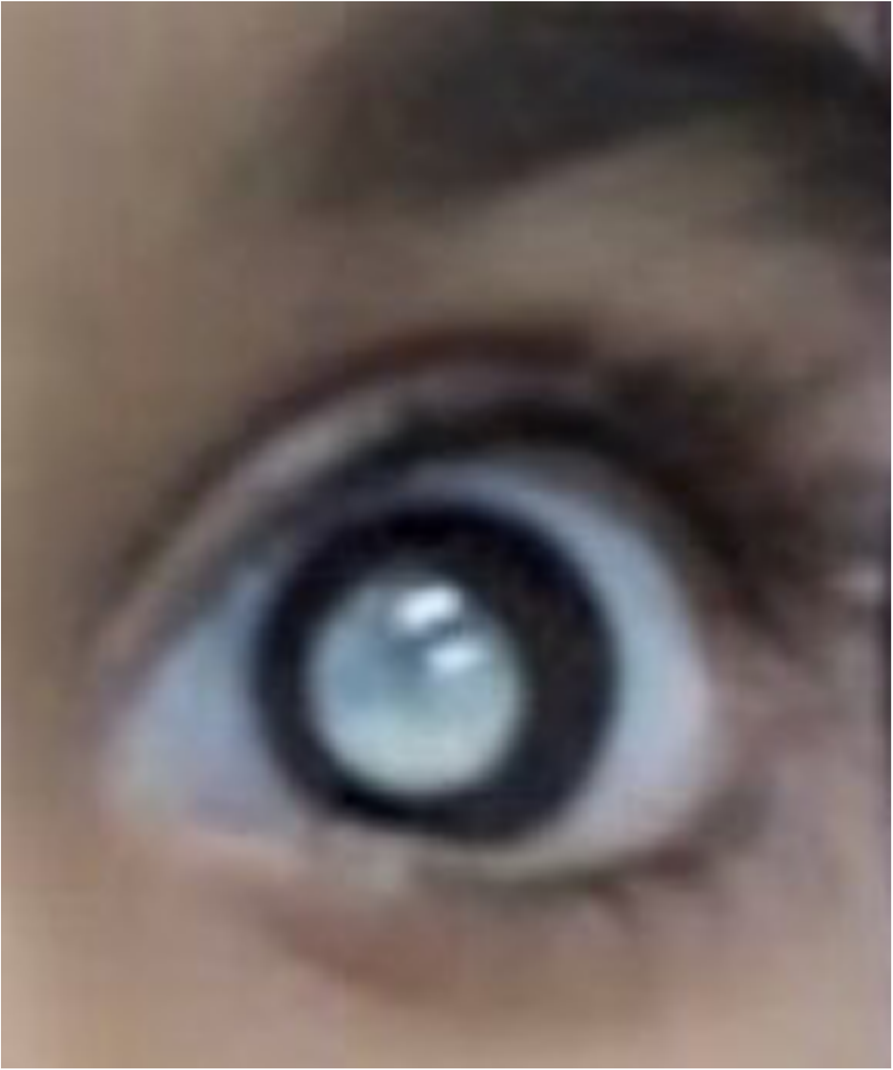
Leukocoria in left eye

Physical examination of the patient showed that the weight and height were below the 3rd percentile and mid-upper arm circumference <115 mm. The patient was ill-appearing. Her peak heart rate was 120/min and her blood pressure was 70/40 mmHg. Leukocoria and glaucoma were detected in left eye (Fig. 4). The patient had hypotonia and her muscle strength in bilateral lower and upper extremities was 3/5. The liver and the spleen were not palpable and the percussion over Traube’s space produced dull sounds during the abdominal examination.

Laboratory test results were: hemoglobin (Hb): 6.5 gr/dL, hematocrit (Hct): 21%, thrombocyte count: 292000/mm3, white blood cell (WBC) count: 2010/mm^3^, international normalized ratio (INR): 1.3, alanine aminotransferase (ALT): 113 U/L, aspartate aminotransferase (AST): 115U/L, gamma-glutamyl transferase (GGT): 245 U/L, albumin: 3.21 gr/dL. The metabolic studies did not show any abnormalities and the levels of viral markers, alpha 1 antitrypsin, alpha fetoprotein, ceruloplasmin, autoantibody and tissue transglutaminase were normal.

**Fig. 5:**
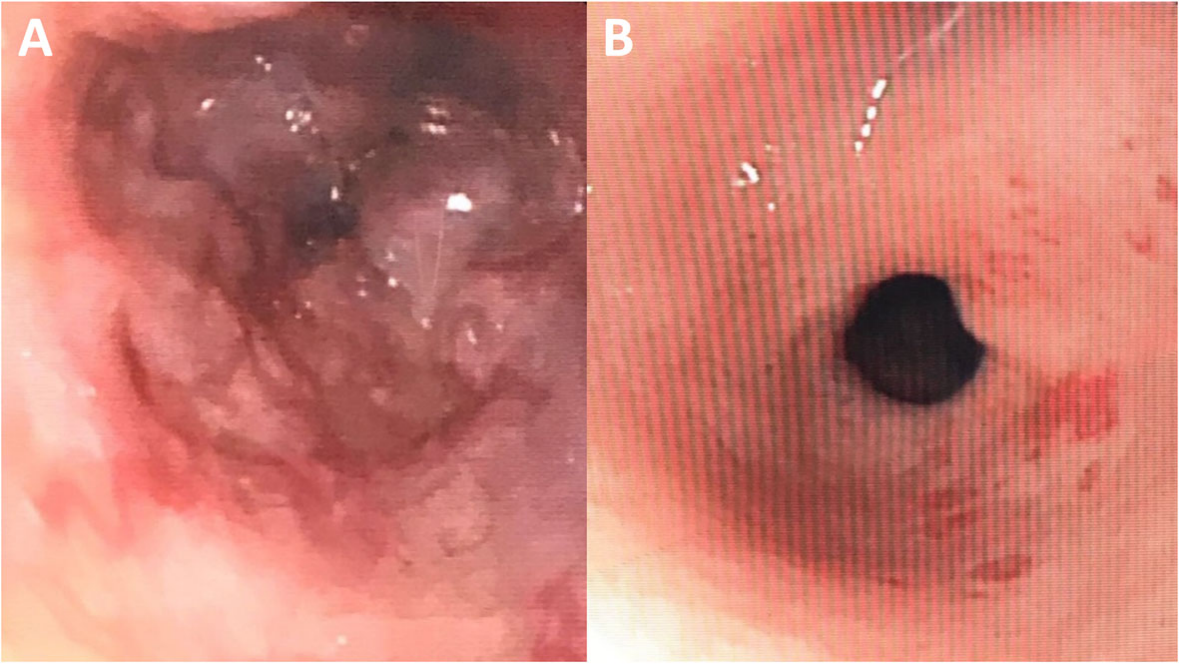
Gastrointestinal endoscopy images **A**: Esophageal varices **B**: Vascular telangiectasia near pyloric antrum

Abdominal ultrasonography showed linear echogenicity and heterogeneity in the liver. Abdominal doppler ultrasonography suggested portal hypertension. An upper and lower gastrointestinal endoscopy revealed folded vascular appearance reminiscent of esophageal varices, as well as vascular telangiectasia in the pyloric antrum, duodenum and colon (Fig. 5). A liver biopsy revealed portal fibrosis.

The patient was initially received nothing-by-mouth diet and intravenous (IV) proton pump inhibitors. The severe gastrointestinal bleeding of the patient was later stopped after receiving IV octreotide and erythrocyte transfusion. The patient kept being treated with propranolol, ursodeoxycholic acid and anticonvulsant. The patient was followed in the pediatric gastroenterology, neurology, and eye clinics. She continued to have intermittent gastrointestinal system bleeding and had severe malnutrition; therefore, the patient was admitted to the emergency department and hospitalized multiple times. The patient was treated with tranexamic acid when she had mild bleeding and received IV octreotide when the bleeding was severe. The patient later was admitted to the intensive care unit due to multi-organ failure secondary to severe gastrointestinal system bleeding, and for that reason an emergency endoscopy could not be performed. She received IV octreotide and erythrocyte transfusion again; however, her condition did not improve, and she eventually died.

## Discussion and conclusions

CRMCC is a rare disease with autosomal recessive pattern occurring due to a mutation in CTC1 gene which is located on chromosome 17p13.1 region and responsible for telomere replication [1–3]. CRMCC can lead to a variety of symptoms including retinal involvement, intracranial calcifications, leukodystrophy, restriction in prenatal and postnatal growth, changes in bone and skeletal system, osteopenia, portal hypertension and life-threatening gastrointestinal system bleeding [1].

The patients with Coats plus syndrome are characterized by IUGR. IUGR is generally followed by restriction in prenatal and postnatal growth but there are cases with normal growth pattern [4]. Our patient was born with IUGR and the physical examination showed that the weight and the height were below 3rd percentile and mid-upper arm circumference <115 mm, which indicates severe malnutrition.

Ocular symptoms may occur with retinal telangiectasia, subretinal exudate, vitreous bleeding, angiomatous nodules, glaucoma, unilateral or bilateral blindness [4]. Neurologic symptoms can appear in different forms such as epilepsy, ataxia, spasticity, dystonia depending on the involved part of the brain [4]. The changes in brain tissue and signaling in brain alongside cysts and calcifications can be detected using Neuroradiological imaging of CRMCC [2]. At the age of 1 month, our patient was diagnosed with vision loss in her left eye and was treated with laser photocoagulation. There were leukocoria and glaucoma in the left eye. The cranial MRI was performed in the pediatrics neurology due to convulsions at 6 months of age and it showed diffuse symmetric calcifications, hemorrhage, dilated lateral ventricles, septated cystic lesions in the area extending from the roof of the third ventricle to the lateral ventricle and hemorrhage in the globe in left orbit.

Life-threatening symptoms such as gastrointestinal system bleeding and portal hypertension can be observed in patients with Coats plus syndrome. Liver failure and gastrointestinal bleeding play major roles in mortality and morbidity and inflammatory changes, abnormal vascularization, telangiectatic vessels can be detected in liver biopsy. [4–6]. In the current literature, embolization and hormonal therapy have been used to stop gastrointestinal bleeding with CRMCC[6–8]. Gastrointestinal endoscopies showed our patient had and esophageal varices and vascular telangiectasia throughout the gastrointestinal tract. In addition, a liver biopsy showed our patient had portal fibrosis. Initially, treatment with IV octreotide resolved the bleeding; however, the patient was later admitted to the intensive care unit and eventually died because of multi-organ failure secondary to severe gastrointestinal bleeding.

In summary, Coats plus syndrome can cause serious life-threatening gastrointestinal bleeding. There are only few reported cases and there is no established treatment approach for gastrointestinal bleeding with Coats plus syndrome [7]. This report presents a case of Coats plus syndrome as a rare cause of gastrointestinal bleeding and portal hypertension.

## Data Availability

The datasets used and/or analyzed during the current study are available from the corresponding author on reasonable request.

## Abbreviations

CRMCC: cerebroretinal microangiopathy with calcifications and cysts
CTC1: encoding conserved telomere maintenance component 1
IUGR: intrauterine growth restriction
MRI: magnetic resonance imaging
Hb: hemoglobin Hct: hematocrit WBC: white blood cell INR: international normalized ratio ALT: alanine aminotransferase AST: aspartate aminotransferase GGT: gamma-glutamyl transferase IV: intravenous
